# Chlorpromazine induced acute cytolytic hepatitis: Case report and a literature review

**DOI:** 10.1192/j.eurpsy.2025.2082

**Published:** 2025-08-26

**Authors:** R. Jbir, F. Guermazi, N. Bouattour, D. Mnif, F. Cherif, I. Bouaziz, R. Atheymen, K. Ksouda, K. Zghal, I. Baati, J. Masmoudi

**Affiliations:** 1 psychiatry ‘A’ department; 2 Psychiatry ‘A’ deprtment, Hedi Chaker university hospital; 3 Regional Pharmacovigilance Center, faculty of medicine of sfax, Sfax, Tunisia

## Abstract

**Introduction:**

Chlorpromazine is historically the first antipsychotic drug. It has played a decisive role in the development of neuropsychopharmacology. However, it can induce adverse effects, sometimes fatal.

**Objectives:**

To study the relationship between acute cytolytic hepatitis and treatment with chlorpromazine.

**Methods:**

We report the case of a patient treated for schizophrenia who developed an acute cytolytic hepatitis after taking chlorpromazine.

**Results:**

We report the case of a 22-year-old woman with no significant medical history, has been diagnosed with schizophrenia who was admitted to our department for physical hetero-aggressiveness.

The clinical examination and laboratory tests performed on admission were normal. The patient was treated with 15 mg of haloperidol and 30 mg of diazepam and 50 mg of chlorpromazine.

On the twelfth day, the patient developed a fever of 38.4°C and abdominal pain. Laboratory tests revealed predominant hepatic cytolysis with elevated ALAT (ASAT: 372 IU/L; ALAT: 463 IU/L), with hepatic cholestasis (PAL: 156 IU/L; GGT: 238 IU/L). Total bilirubin and prothrombin levels were normal with no evidence of jaundice or pruritus

The ALAT/PAL ratio was 8.8 > 5, indicating cytolytic hepatitis. The rest of the clinical and biological examination was completely normal.

Drug-induced hepatitis was suspected and the three drugs were stopped after consultation with the regional pharmacovigilance center in Sfax. The biological control showed a decrease in transaminases (ALAT=5.4*N, ASAT=1.3*N) 3 days after stopping the treatment. Viral serology (hepatitis A,B and C) was negative and liver ultrasound showed hepatomegaly suggestive of drug-induced hepatitis.

Haloperidol-induced cytolysis was initially suspected, and the prescription of chlorpromazine was authorized. Due to the patient’s instability, she was treated with chlorpromazine at a dose of 25 mg*3/d. Twenty-four hours after administration of this neuroleptic, the patient presented a worsening of hepatic status: ALAT=20*N, ASAT=14.4*N, PAL=2.6*N, GGT=19*N. The ALAT/PAL ratio was 7.6, indicating cytolytic impairment requiring immediate discontinuation of chlorpromazine.

The pharmacovigilance investigation blamed chlorpromazine and contraindicated its use. The evolution was characterized by an improvement of the hepatic balance three days after discontinuation of chlorpromazine.

Normalization of transaminases was achieved within 3 weeks. GGT and PAL were normalized after 2 months of discontinuation.

Chlorpromazine was classified as probable (C2S3I3B3(R+)). The score was calculated according to the French Bégaud method.

**Conclusions:**

Edema is not a common side effect of typical antipsychotics. However, it is crucial to emphasize that this effect can occur. Clinicians are advised to watch for edema in all patients taking antipsychotics.

**Disclosure of Interest:**

None Declared

